# Supplementary material for: Dietary Insect Powder Protein Sources Improve Protein Utilization by Regulation on Intestinal Amino Acid-Chemosensing System
Source: Animals (Basel). 2020 Sep 7;10(9):1590. doi: 10.3390/ani10091590 (PMC7552256; doi:10.3390/ani10091590)
Supplement: Supplementary file 1 [file animals-10-01590-s001.pdf]

Table S1. Primers used in this study

Gene[1]	Accession No.	Primer, 5′–3′	Size, bp
β-actin	XM_021086047.1	F: CTGCGGCATCCACGAAACT	147
		R: AGGGCCGTGATCTCCTTCTG	
TRPV1	XM_013981216.2	F:ATTACACCCGTGGCTTCCAG	199
		R: CAGACAAGCCCTCGACACTT	
FFAR3	NM_001315601.1	F:ACCGTCTATCTCACGTCCCT	87
		R:CCGGGTCTTGTACCAGAGTG	
TRPM5	XM_021082617.1	F: GAACTGATCTTCCTGGCCCC	105
		R: CTTTTGAGGCCACAAGCCAC	
mGluR5	XM_021102332.1	F: GGGGAAACCCTAAGCTCCAAT	516
		R: GGAGTTGAGCTCGCTGATGT	
T1R1	XM_013988748.2	F: TCAACGTGTTTGCCATGCTG	367
		R: AAGGAGGCTCTGCCCATTTC	
PLCβ2	XM_021097783.1	F: GCTTGGCCAGGTAGCTGATA	165
		R: GGACAGACCCCCATCCAAAG	
GPRC6A	XM_021089187.1	F: GCCGGGATTTGTCCACAGTA	160
		R: TGAGCTCCCTCCATCCATGA	
PAT1	NM_203174.3	F: GGAGCGACATGTTTGAGTGG	323
		R: TGATCTGCACAATAACTTTTTAGGT	
PAT2	NM_202714.4	F: TGCAGAAAAGCGTCCAAATGA	540
		R: TCTCTGCCTAGGCCATCCTT	
Y+LAT1	XM_013978228.1	F: GAGTGCCAGAACACAAACGA	116
		R: TCCTCCATCTTCCAAATCCA	
SNAT2	NM_001317081.1	F: CTGAGCAATGCGATTGTGGG	615
		R: ACGTGGTCGGCAAGAATCAT	
GCN2	NM_001339970.1	F: GGCCTTTAAACTCATTGGCGT	628
		R: GATTGCTATTTGCGGAGGCG	
MAP4K3	XM_021088335.1	F: AGACTGCTTGGATCTGACAGG	552
		R: TGTGCTGTTTCATTGCCAGC	
PIK3C3	NM_001012956.2	F: GTCCGATGAAGAGGCAGTCC	77
		R: CTACCACCGCAGCAAACAGA	
S6K1	XM_001693507.1	F: CTCAATGGCGGACACCTCTT	589
		R: CCATGTTGCATGTGACGGTG	

[1]*TRPV1*, The transient receptor potential cation channel subfamily V member 1; *FFAR3,* free fatty acid receptor 3; *TRPM5*, transient receptor potential cation channel subfamily M member 5; *mGluR*, metabotropic glutamate receptors; *T1R1*, taste 1 receptor member 1; *PLCβ2*, Gβγ-mediated phospholipase C β2; *GPRC6A*, G protein-coupled receptor family C group 6 member A; *PAT1*, proton-coupled amino acid transporter 1; *PAT2*, proton-coupled amino acid transporter 2; *Y+LAT1*, L-type amino acid transporter 1; *SNAT2*, sodium dependent neutral amino acid transporter 2; *GCN2*, general control nonderepressible 2; *MAP4K3*, mitogen-activated protein 4 kinase 3; *PIK3C3*, phosphatidylinositol 3-kinase catalytic subunit type 3; *S6K1*, ribosome protein subunit 6 kinase 1.
